# Chromatographic fingerprinting and antibiofilm effect of *Ziziphus jujuba* fraction on *Pseudomonas aeruginosa*

**DOI:** 10.1186/s13568-025-01886-6

**Published:** 2025-05-21

**Authors:** Mohamed Turkey, Jilan A. Nazeam

**Affiliations:** 1 Microbiology and Immunology Department, Faculty of Pharmacy, October 6 University, Six of October City, 12585 Egypt; 2 Pharmacognosy Department, Faculty of Pharmacy, October 6 University, Six of October City, 12585 Egypt

**Keywords:** Biofilm formation, Liquid chromatography, Mass spectroscopy, *Pseudomonas aeruginosa*, Standardized fraction, Virulence genes, *Ziziphus jujuba* seeds

## Abstract

*Pseudomonas aeruginosa* represents a critical global health threat, particularly affecting immunocompromised individuals, as well as patients with wounds and burn injuries. The increasing prevalence of multidrug-resistant (MDR) *P. aeruginosa* strains has significantly reduced the efficacy of conventional antimicrobial therapies, underscoring the urgent need for new, effective therapeutic alternatives. Plant-derived secondary metabolites have emerged as promising candidates due to their diverse bioactivities and favorable safety profiles. This study investigated the antimicrobial and anti-virulence potential of purified aqueous fractions of *Ziziphus jujuba* (ZJ) seeds against MDR *P. aeruginosa* clinical isolates. LC–ESI–MS/MS-MRM fingerprinting identified 33 compounds, including five predominant phenolics: 3,4-dihydroxybenzoic acid, gallic acid, syringic acid, chlorogenic acid, and ferulic acid. One hundred clinical isolates were evaluated for antibiotic sensitivity and biofilm-forming ability. The ZJ fraction exhibited potent antibacterial activity, with a minimum inhibitory concentration (MIC) of 1.56 mg/ml and significantly inhibited biofilm formation by approximately 70%. Additionally, quantitative real-time PCR showed a marked downregulation the key quorum-sensing genes *lasI* (45%), *rhlI* (42%), and *rhlR* (34%) (*p* ≤ 0.05). These findings reveal, for the first time, that the aqueous fraction of *Z. jujuba* seeds not only inhibits bacterial proliferation, but also attenuates biofilm formation and virulence gene expression in MDR-*P. aeruginosa.* These results highlight the potential of ZJ fraction as a promising plant-based antimicrobial agent. Further in vivo investigations and mechanistic studies are warranted to validate its clinical applicability and therapeutic efficacy.

## Introduction

Chronic microbial infections represent a substantial challenge to global public health, largely attributable to the rise of multidrug-resistant (MDR) pathogens (Ho et al. 2024). The World Health Organization (WHO) reports that antibiotic resistance is reaching critical levels and is anticipated to become a leading cause of mortality by 2050, with an estimated 10 million deaths annually (Kraker et al. 2016). MDR-pathogens constitute a major concern for patients in healthcare settings and individuals with weakened immune defenses, leading to elevated rates of disease and mortality among these susceptible groups (Christaki et al. 2020; Galal et al. 2021; Abu-Hussien et al. 2024).

*Pseudomonas aeruginosa*, recognized for its opportunistic characteristics, is a Gram-negative bacterium that poses significant concern as a multidrug-resistant (MDR) pathogen, frequently implicated in healthcare-acquired infections (HAI) (Magryś et al. 2021). The 2024 Bacterial (Magryś et al. 2021). The 2024 Bacterial Priority Pathogens List (BPPL) published by the World Health Organization (WHO) highlights highly resistant *P. aeruginosa* as a critical pathogen that demands immediate global attention. This emphasizes the urgent need for innovative strategies to combat the rising challenge of antimicrobial resistance (AMR) (Moustafa et al. 2024).

Multidrug-resistant *P. aeruginosa* is implicated in a wide range of infections, including endocarditis, urinary tract infections, burns, and wound infections (Sanford and Gallo 2013; Mehrad et al. 2015). A key factor in *P. aeruginosa* pathogenicity is its ability to form biofilms. According to the U.S. At the National Institute of Health (NIH), approximately 80% of chronic bacterial infections are associated with biofilm production (Jamal et al. 2018).

Biofilms are intricate microbial assemblages encapsulated within a self-generated extracellular polymeric matrix consisting of exopolysaccharides (EPS), proteins, and lipids (Su and Hassett 2012; Gomaa et al. 2024). This matrix acts as a protective barrier that impedes antibiotic penetration, allowing bacteria to withstand antimicrobial concentrations up to 1,000 times higher than those effective against free-floating (planktonic) cells (Vestby et al. 2020). This phenomenon enhances bacterial fortification and hampers antibiotic dissemination, thus promoting antimicrobial resistance (Reynolds and Kollef 2021). Biofilms significantly contribute to the development of wounds, burn infections, and inflammatory responses, particularly in healthcare-associated infections (HAI) (Mehrad et al. 2015; Hassett et al. 2010; El-Sayed et al. 2020), as well as nosocomial infections (Khadraoui et al. 2022; Mohamed et al. 2024).

Various strategies have been proposed to inhibit biofilm formation and mitigate its effect on the progression of infectious diseases (Hurley et al. 2012). Key regulatory genes involved in *P. aeruginosa* biofilm formation and virulence include *lasI*, *lasR*, *rhlI*, and *rhlR* (Lee & Zhang 2015; Wu et al. 2001). Targeting these genes has been identified as a new therapeutic strategy for mitigating MDR *P. aeruginosa* infection (Nogueira et al. 2017; Craft et al. 2019; Parham et al. 2020).

In light of the limitations associated with conventional antibiotics and the increasing prevalence of antibiotic-resistant bacterial pathogens, there is a growing interest in exploring plant-derived compounds as alternative antimicrobial agents (Rezk et al. 2022; Rossi et al. 2022). Medicinal plants have historically been sources of bioactive compounds with antibacterial properties, offering promising solutions for addressing MDR infections (Silva et al. 2021, (Clinical and Laboratory Standards Institute (CLSI) 2022).

*Ziziphus jujuba*, a member of the Rhamnaceae family (Yu et al. 2012, Mehrad et al. 2015), has a long history of use in traditional medicine and is currently marketed as a nutritional supplement (Hassett et al. 2010; Agrawal et al. 2023). The plant contains a wide variety of phytochemicals, including phenolics, tannins, flavonoids, triterpenic acids, cerebrosides, saponins, and minerals, which are responsible for its diverse biological activities (Hurley et al. 2012; Su & Hassett 2012; Lee & Zhang 2015; Ji et al. 2017; Rezk et al. 2022; Agrawal et al. 2023). Several studies have reported the antimicrobial properties of *Z. jujuba* extracts against a range of bacterial pathogens including *Escherichia coli*, *Staphylococcus aureus* (Bibi et al. 2014; Beg et al. 2016), *Klebsiella*spp., *E. coli* (Alhassan et al. 2019), *Pseudomonas aeruginosa*, *Bacillus pumalis*, *Salmonella typhi*, *Enterobacter aerogenes*, and *Staphylococcus epidermidis* (Ahmad et al. 2011).

While these findings are acknowledged, the influence of *Z. jujuba* on the expression of virulence genes in multidrug-resistant *P. aeruginosa* has yet to be investigated. At present, there is a paucity of research investigating the capacity of *Z. jujuba* to inhibit biofilm formation through the modulation of quorum sensing regulatory genes. This study aims to address a significant gap in the availability of plant-derived anti-virulence agents. It examines the impact of a purified aqueous extract of *Z. jujuba* seeds (ZJ) on the expression of key quorum sensing genes (lasI, rhlI, and rhlR) and biofilm formation by MDR *P. aeruginosa*. Additionally, the research aims to chemically standardize the bioactive ZJ fraction using LC–MS/MS-MRM to provide insights into its potential as an alternative therapeutic agent for drug-resistant bacterial infections.

## Materials and methods

### Plant material

*Ziziphus jujuba* seeds were obtained from the Egyptian market and were later identified by a knowledgeable botanist at the Agricultural Museum in Giza, Egypt. The checked samples were placed on October 6 University, Faculty of Pharmacy, the herbarium of the Pharmacognosy Department (code ZJ-01).

### Bacterial isolates

A total of one hundred *P. aurgenosa* isolates from clinical specimens were isolated from February 2023 to January 2024 all from burn and wound patients who were admitted to the Microbiology Laboratory, from different hospitals across Egypt and mainly from October 6 University Hospital. Cetrimide agar was used, isolates were cultivated, and species were identified by a systematic microbiological panel, including colony morphology (green pigments), Gram staining (gram-negative rods), and biochemical reactions (oxidase-positive and citrate-positive). After phenotypic identification, we performed further identification using an automated Vitek 2 Compact system (bioMérieux, France) using the protocol attached. Isolates and samples were placed in Luria–Bertani glycerol and kept at − 80 °C for different investigations.

*P. aurgenosa* clinical isolate C1 isolated from burn and wound patients were deposited in the Culture Collection of Ain Shams University (coded CCASU-2024–74) of the World Data Centre for Microorganisms (WDCM) (https://doi.org/10.12210/ccinfo.1186). This isolate was identified by sequencing 16S ribosomal RNA and was deposited in NCBI GenBank (Accession code, PQ527912). The *P. aeruginosa* clinical isolates included in this study represent diverse sampling of strains from various infection sites and antimicrobial resistance profiles. The sample size was chosen based on previous research into antimicrobial resistance patterns in clinical isolates, which typically ranges from 50 to 150 isolates to ensure reliable statistical power and pattern recognition. Isolates were collected from burn wounds, surgical sites, and abscesses across multiple healthcare facilities to capture a broad clinical spectrum. The inclusion criteria required confirmation of P. aeruginosa isolates through both traditional microbiological methods and the Vitek 2 Compact system. The exclusion criteria were duplicate strains from the same patient and those with incomplete antibiograms. This approach enhances the reliability of the observed biofilm formation trends and the response to plant extracts across genetically and phenotypically diverse strains.

### Preparation of aqueous *Ziziphus jujuba* fraction

*Ziziphus jujuba* seeds (250 gm) were boiled in distilled water at 100 ^◦^C for three hours. The aqueous extract was concentrated to 1/10 volume of by rotary evaporation. Cold absolute ethanol was then added for purification and precipitation of high-molecular-weight compounds (Nazeam et al. 2020). The aqueous filtrate was concentrated using a rotary evaporator to obtain a purified fraction (ZJ). The fraction was stored at − 20 °C in a securely closed glass vessel.

### Liquid chromatography–electrospray ionization–tandem mass spectrometry using multiple-reaction monitoring (MRM) mode

The samples were analyzed using liquid chromatography–electrospray ionization–tandem mass spectrometry (LC–ESI–MS/MS-MRM) with an Exion LC AC system for separation and an SCIEX Triple Quad 5500 + MS/MS system equipped with electrospray ionization (ESI) for detection. The separation was performed with an Ascentis® Express 90 Å C_18_ Column (2.1 × 150 mm, 2.7 µm). The mobile phase consisted of two eluents: A, 5 mM ammonium format, pH 8; and B, acetonitrile (LC grade). The mobile phase gradient was programmed as follows: 5% B for 0–1 min, 5–100% B for 1–20 min, 100% B for 20–25 min, 5% at 25.01, 5% for 25.01–30 min. The flow rate was 0.3 ml/min, and the injection volume was 5 µL. For MS/MS analysis, the negative ionization mode was applied with a scan (EMS-IDA-EPI) from 100 to 1000 Da for MS1 with the following parameters: curtain gas: 25 psi; IonSpray voltage: − 4500; source temperature: 500 °C; ion source gas 1 and 2 were 45 psi and from 50 to 1000 Da for MS2 with a delustering potential of: − 80; collision energy: − 35. Compounds were identified using MS-DIAL with respect to the library.

### Phenotypic detection of biofilm formation

Biofilm formation was quantified. Bacterial broth was incubated overnight, the cultures were adjusted to a McFarland turbidity of 0.5, and the broth was diluted to 1:100 in LB broth. A bacterial suspension (200 μL) was added to three wells of a sterile 96-well plate and incubated at 37 °C for 24 h, considering that a well containing broth only was used as a negative control. A standard strain was used as the positive control (*P. aeruginosa* ATCC 12924). The contents of each well were then aspirated and cleaned. A 200μL sterile phosphate-buffered saline was added to wash the wells, and the plates were placed in a shaker to remove all non-attached bacteria. Adherent bacteria were fixed by adding 200 μL 99% methanol to each well. The wells were then stained with 200 μL 2% Hucker crystal violet for 15 min. Additional stain was washed off by immersing the plate in distilled water; the dye adherent to the attached cells was resolubilized by adding 200 μL of 33% (v/v) acetic acid to each well. Eventually, the optical density was assessed at 595 nm using an ELISA reader (TECAN, Switzerland); a negative control of 33% acetic acid was used. Quantification was performed according to the method described by Stepanovic et al. (2000) using microtiter plates.

The test was performed in triplicate for each isolate, and the mean results of the three wells were calculated. The ability to adhere to the tested isolates was also assessed. There were 4 clases of biofilm production summarized as: non-adherent (OD ≤ ODc), weakly adherent (ODc < OD ≤ 2ODc), moderately adherent (2ODc < OD ≤ 4ODc), or strongly adherent (OD > 4ODc), based upon the calculated cut-off OD (ODc) (three standard deviations above the mean OD of the negative control).

### Molecular characterization

The clinical isolate (C1) with the highest antibiotic resistance and the strongest biofilm production was further identified by 16S rRNA amplification and sequencing as follows: overnight broth cultures of the isolated strains were centrifuged for 15 min at ambient temperature. The supernatants were discarded, and the pellets were used for bacterial DNA extraction using the GeneJETTM PCR Purification kit (Thermo Scientific) according to the manufacturer’s instructions. The dignity of the nucleic acids was evaluated by 1% agarose gel electrophoresis containing ethidium bromide, and DNA was stored at 20 C. PCR amplification was performed using the universal primers 16S-27F (5′AGAGTTTGATCCTGGCTCAG3′) and 16S-1492R (5′TACGGTTACCTTGTTACGACTT 3′) in a thermal cycler (Applied Biosystems). Each PCR reaction was conducted using 25 µL of Maxima® Hot Start PCR Master Mix (2X), 0.4 µL of each primer (10 µM), 5 µL of template DNA, and 18 µL nuclease-free water. A reaction without DNA was used as a negative control. The 16S rRNA gene sequences were compared with those in the NCBI GenBank database using the Blastn tool to identify the two isolates. The 16S rRNA sequences were submitted to the NCBI database under accession number PQ527912.

### Antimicrobial susceptibility testing

Antibiotic susceptibility patterns of 100 Pseudomonas isolates were assessed using the Kirby-Bauer disc diffusion method, using a sterile loop, 3–5 colonies were selected from each isolate and suspended in 5 mL of sterile 0.9% saline to prepare a standardized bacterial suspension equivalent to 0.5 McFarland turbidity. Sterile Mueller–Hinton agar (MHA) plates were uniformly inoculated with the bacterial suspension using sterile swabs. Antibiotic discs were carefully placed on the surface of the inoculated plates, which were then incubated at 37 °C for 24 h. After incubation, the diameters of the zones of inhibition around each disc were measured.

### Screening for the antibacterial effect of ZJ fraction

The cup-plate technique was used to assess the antibacterial effects of the aqueous fraction of *Z. jujuba* seeds. All cultures of *P. aeruginosa* isolates were adjusted to 0.5 McFarland then streaked onto MHA. The previously prepared aqueous plant fraction was adjusted to 100 mg/mL and 200 μL was added to each cup. All plates were incubated at 37 °C for 24 h.

### Minimum inhibitory concentration assay (MIC)

MICs of the aqueous herbal fraction was determined using broth microdilution method.

A Flat-bottomed 96-well microtiter plate was implemented with three strong biofilms producing MDR *P. auregenosa* isolates (C1, C2, and C3) in Muller Hinton Broth (MHB). *Ziziphus* seed aqueous fraction was prepared at a concentration of 100 mg/mL, and a two-fold dilution of the herbal aqueous preparation of 2 folds were done at a concentration of 50 mg/mL and ending at 0.049 mg/mL. 100μL of bacterial cultures was inoculated into 100 μL of the aqueous herbal fraction at different concentrations, and the plate was incubated at 37 °C for 24 h. The fraction was tested in triplicate According to the CLSI 2022, and the minimum inhibitory concentrations were estimated using the microdilution method of the Clinical and Laboratory Standards Institute (CLSI) 2022).

### Effect of a sub-inhibitory concentration of ZJ fraction on biofilm-formation

Performed by microtiter plate technique, which is previously illustrated the biofilm inhibitory action of the herbal aqueous fraction to the selected isolates was measured at half MIC; plane wells of aqueous herbal fraction were used as controls. The degree of biofilm downregulation was calculated using the following formula:

$$\text{\% Inhibition}=\frac{\text{OD control}-\text{OD sample}}{\text{OD control}}\times 100$$

### Transcriptional analysis

*Pseudomonas aeruginosa* isolates (C1, C2, and C3) were selected and grown on Luria–Bertani agar and then inoculated in liquid broth with or without the sub-MIC of the ZJ fraction. The test plates were then incubated in a shaker at 225 rpm/37 ℃. After 6–8 h of shaking in an incubator shaker, approximately 10 mL was taken (as bacteria were in the stationary phase), and 10 mL of each inoculum was centrifuged for 5 min at 5000 rpm. The supernatant was discarded, and the pellets were saved for sequential assays.

### RNA extraction and reverse transcription

Total RNA purification was performed using a ready-to-use Invitrogen™ TRIzol™ reagent (Simms et al. 1993). A Reverse Transcription Kit (Qiagen, USA) was used to reverse-transcribe 1 μg of total RNA in a two-step reaction. Genomic DNA (gDNA) contamination was excluded using the Wipeout buffer.

### Gene expression analysis using real-time PCR for quantification

Specific primer pairs were used to amplify total cDNA (30 ng) (Table 1) and Maxima SYBR Green/Fluorescein qPCR Master Mix (2X, Thermo Scientific, Waltham, MA, USA). Quantitative real-time PCR was performed in triplicate for the selected samples. Rotor-Gene Q MDx (Qiagen, USA) performed amplification, automatically collected the data, and then analyzed the value of the threshold cycle, which was normalized to the average Ct value of the housekeeping gene. The 2^ − ΔΔCT^ method was used to calculate the relative gene expression fold change, which was standardized to the reference gene.

**Table 1:** List of primer sequences used for quantitative real-time PCR (qRT-PCR)

Gene	Primer direction	5′- 3′ sequence	Reference
*lasI*	Forward	GTGACGGTAACCACCGTAGG	4
	Reverse	CTGGGTCTTGGCATTGAGTT	
*rhlI*	Forward	AAGGACGTCTTCGCCTACCT	4
	Reverse	GCAGGCTGGACCAGAATATC	
*rhlR*	Forward	CATCCGATGCTGATGTCCAACC	
	Reverse	ATGATGGCGATTTCCCCGGAAC	
*5S rRNA*	Forward	TGACGATCATAGAGCGTTGG	4
	Reverse	GATAGGAGCTTGACGATGACCT	

### Statistical analysis

Statistical analysis was conducted using one-way analysis of variance (ANOVA) to assess significant differences between the control and tested isolates, with a significance threshold set at *p* ≤ 0.05.

## Results

### LC–ESI–MS/MS analysis

**Fig. 1:**
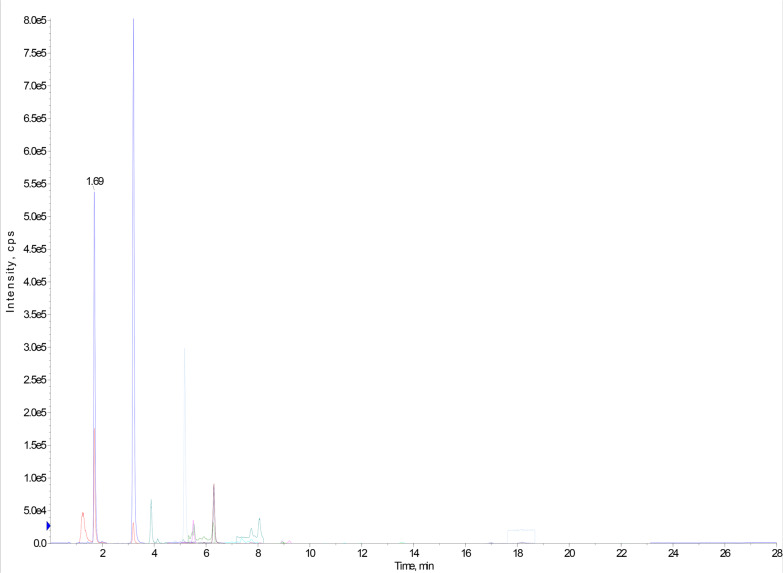
LC–ESI–MS/MS chromatograms obtained in MRM mode of phenolics and flavonoids in the aqueous fraction of *Z. jujuba*

The detection and quantification of phenolic compounds in the *Ziziphus jujuba* (ZJ) fraction were conducted using liquid chromatography-electrospray ionization tandem mass spectrometry. In MRM mode, LC–ESI–MS/MS chromatograms were used to obtain a mixture of standard phenolic and flavonoid compounds. Standardized fingerprinting analysis identified five major phenolic compounds; 3,4-dihydroxybenzoic acid (99.03 ug/g) followed by gallic acid (60.56 ug/g), syringic acid (53.44 ug/g), chlorogenic acid (11.06 ug/g), and ferulic acid (2.5 ug/g). Based on the inserted parent ions (Q1), product ions (Q3), retention times, and reported literature, the fraction compounds were identified, as presented in Table 2 and Fig. 1.

**Table 2:** MRM parameters and identification of phenolic compounds in aqueous ZJ by LC–ESI–MS/MS

ID	Conc. (ug/g)	Q1 (m/z)	Q3 (m/z)	RT (min)	CE (V)	CXP (V)	DP (V)
Gallic acid	60.56	168.9	124.9	1.6	− 30	− 11	− 110
3.4-dihydroxybenzoic acid	99.03	152.9	109	3.2	− 40	− 5	− 75
Chlorogenic acid	11.06	353	191	5.1	− 23	− 10	− 60
Syringic acid	53.44	196.9	181.9	6.3	− 12	− 5	− 30
Ferulic acid	2.50	192.8	133.9	8.9	− 16	− 5	− 25

Collision energy (CE). Collision cell exit potential (CXP) declustering potential (DP)

LC–ESI–MS/MS scanning analysis revealed the presence of 33 phenolic compounds (Table 3). Among these, caffeic acid, chlorogenic acid, gallic acid, ferulic acid, quinic acid, syringic acid, malic acid, maleic acid, sucrose, kaempferol-3-glucuronide, naringenin-7-O-glucoside, 7-hydroxy-4-methylcoumarin, glycyl-L-proline, and l-arginine have been identified in *Z. jujuba*. The other 19 compounds are reported here for the first time. This study is the first to investigate phenolic compounds in *Z. jujuba* seeds using LC–ESI–MS/MS-MRM analysis.

**Table 3:** LC–ESI–MS/MS scanning for identification of phenolic compounds in ZJ fraction using negative ion mode

ID	Metabolite name	Chemical formula	RT (min)	Exact mass	[M-H]-	S/N	References
1	Caffeic acid	C_9_H_8_O_4_	0.411	180.0423	179.0341	2.04	Dilek Tepe et al. (2020)
2	HydroxyButyric acid	C₄H₈O₃	0.759	104.0473	102.9950	0.56	Kim et al. (2022)
3	trans-cinnamate	C_9_H_8_O_2_	0.903	148.0524	147.0856	1.99	Redeuil et al. (2009)
4	(-)-Shikimic acid	C_7_H_10_O_5_	1.085	174.0528	173.0312	7.80	Bochkov et al. (2012)
6	Citramalate	C_5_H_8_O_5_	1.174	148.0371	147.0234	7.05	Sugimoto et al. (2021, Umino et al. 2023)
7	N-Isovaleroylglycine	C_7_H_13_NO_3_	1.485	159.1852	158.0173	17.77	Blunden et al. (2006)
8	Gallic acid	C₇H₆O₅	1.692	170.0215	168.9012	25.12	Dilek et al. (2020)
9	4-Hydroxy-3-methoxycinnamaldehyde	C_10_H_10_O_3_	1.648	178.1871	177.0732	29.87	Samuelsen et al. (1986)
10	Sucrose	C₁₂H₂₂O₁₁	1.851	342.1162	340.9944	18.67	Qin et al. (2022)
11	Kaempferol-3-glucuronide	C_21_H_18_O_12_	2.084	462.3631	460.948	34.19	Qin et al. (2022)
12	Taurine	C₂H₇NO₃S	2.190	125.0146	124.006	21.53	Lee., (2015, Mou et al. 2002)
13	3.4-Dihydroxybenzoic acid	C_7_H_6_O_4_	3.215	154.0266	152.9015	19.31	Dilek et al. (2020)
14	Mucic acid	C₆H₁₀O₈	3.601	210.0375	208.9222	20.51	Li et al. (2019)
15	Methyl dihydrojasmonate	C₁₃H₂₂O₃	3.656	226.1568	225.1508	11.50	Huang et al. (2015)
16	Chlorogenic acid	C₁₆H₁₈O₉	5.123	354.0950	353.0923	15.89	Dilek et al. (2020)
17	Syringic acid	C₉H₁₀O₅	6.352	198.0528	196.9351	18.23	Dilek et al. (2020)
18	Delphinidin	C₁₅H₁₁O₇⁺	7.153	303.2467	300.9349	3.20	Husain et al. (2022)
19	Glycyl-L-proline	C_7_H_12_N_2_O_3_	7.259	172.0847	171.2422	10.06	Uddin et al. (2022)
20	Ferulic acid	C_10_H_10_O_4_	8.910	194.1863	192.8234	19.34	Qin et al. (2022)
21	L-Arginine	C_6_H_14_N_4_O_2_	8.946	174.2042	173.1444	3.17	Uddin et al. (2022)
22	Farnesol (mixture of isomers)	C₁₅H₂₆O	9.399	222.1983	221.2474	9.42	Moinuddin et al. (2022)
23	D-(-)-Quinic acid	C₇H₁₂O₆	12.309	192.0633	191.1648	17.64	Wang et al. (2014)
24	(+ -)-Jasmonic acid	C₁₂H₁₈O₃	12.370	210.1255	209.145	15.48	Huang et al. (2015)
25	N-Carbamoyl-L-aspartic acid	C_5_H_6_N_2_O_5_	13.829	176.0433	175.0298	0.89	Panaskar et al. (2023)
26	Daidzein	C₁₅H₁₀O₄	21.257	254.0579	253.2039	10.59	Saha et al. (2020)
27	E-3,4,5'-Trihydroxy-3'-glucopyranosylstilbene	C_20_H_22_O_8_	23.458	406.1263	405.1263	6.45	Teka et al. (2022)
28	D-( +)-Malic acid	C₄H₆O₅	24.113	134.0215	133.0525	0.84	Agrawal et al. (2023)
29	Naringenin-7-O-glucoside	C_21_H_22_O_10_	27.864	434.3975	433.2091	3.04	Qin et al. (2022)
30	7-Hydroxy-4-methylcoumarin	C₁₀H₈O₃	28.441	176.0473	175.0466	5.73	Yan et al. (2015, Tine et al. 2017)
31	3-Hydroxyisovaleric acid	C_5_H_10_O_2_	28.603	118.0629	117.0587	3.68	Recber et al. (2022)
32	Maleic acid	C₄H₄O₄	28.626	116.0109	114.8996	4.32	Agrawal et al. (2023)
33	Urocanic acid	C₆H₆N₂O₂	28.817	138.0429	137.1061	3.65	Lima et al. (2021)

### Bacterial isolates

A total of 100 clinical *P. aeruginosa* isolates were obtained from burn and wound infections in multiple hospitals. All isolates were identified as *P. auregenosa* by traditional microbiological methods and automatically using the Vitek 2 Compact System. The distribution of isolates was 35 (35%) isolates were obtained from chronic surgical wounds, 40 (40%) from burns, and 25 (25%) from abscesses.

### Molecular characterizations

16S rRNA gene sequence analysis of isolates C1, C2, and C3 revealed 99.05% identity with *the P. aeruginosa* strain ATCC 10145 (partial 16S rRNA). The clinical isolate (C1) with the highest antibiotic resistance and the strongest biofilm production was selected for further identification by 16S rRNA amplification and sequencing. The 16S rRNA gene sequences were compared with those in the NCBI GenBank database using the Blastn tool to identify the isolate. Phenotypic identification was confirmed using 16S rRNA gene sequences, which were submitted to the GenBank database (Accession No. PQ527912).

### Biofilm production

Biofilm formation analysis categorized the 100 *P. aeruginosa* isolates as follows: 80% of the Pseudomonas isolates had the ability to produce biofilms. Among them, 35 (35%) isolates were strong biofilm producers, 25 (25%) were intermediate and 20 (20%) were weak producers and only 20 (20%) were non-biofilm producers Table 4 provides the detailed distribution and absorbance readings for the high-, intermediate-, and low-biofilm-forming *P. aeruginosa strains.*

**Table 4:** The percentage of biofilm production in *Pseudomonas aeruginosa* isolates

Biofilm degree	No (%)
High producer	35(35%)
Intermediate producer	25(25%)
Low producer	20(20%)
No biofilm production	20(20%)

### Antimicrobial susceptibility testing for the selected isolates (AST)

Susceptibility testing revealed elevated resistance to multiple antibiotics: ticarcillin, ticarcillin/clavulanic acid, piperacillin, and piperacillin/tazobactam with MIC ≥ 128, and ceftazidime, amikacin, and cefepime with MIC ≥ 64. However, colistin resistance decreased and retained activity with MIC, as shown in Table 5, consistent with the Laboratory Standards Institute (CLSI) 2022 breakpoint.

**Table 5:** Screening of antibiotic activity and susceptibility of the isolates

Antibiotic	Resistant		Intermediate		Sensitive	
	No	%	No	%	No	%
Ticarcillin	100	100	0	0	0	0
Ticarcillin/clavulanic acid	100	100	0	0	0	0
Piperacillin	100	100	0	0	17	17
Piperacillin/tazobactam	80	80	0	0	20	20
Ceftazidime	50	50	0	0	50	50
Cefepime	45	50	5	5	50	50
Lmipenem	40	40	10	10	50	50
Amikacin	30	30	10	10	60	60
Ciprofloxacin	30	30	0	0	70	70
Colistin	0	0	0	0	100	100

### Antimicrobial activity of ZJ fraction on *P. aeruginosa*

**Fig. 2:**
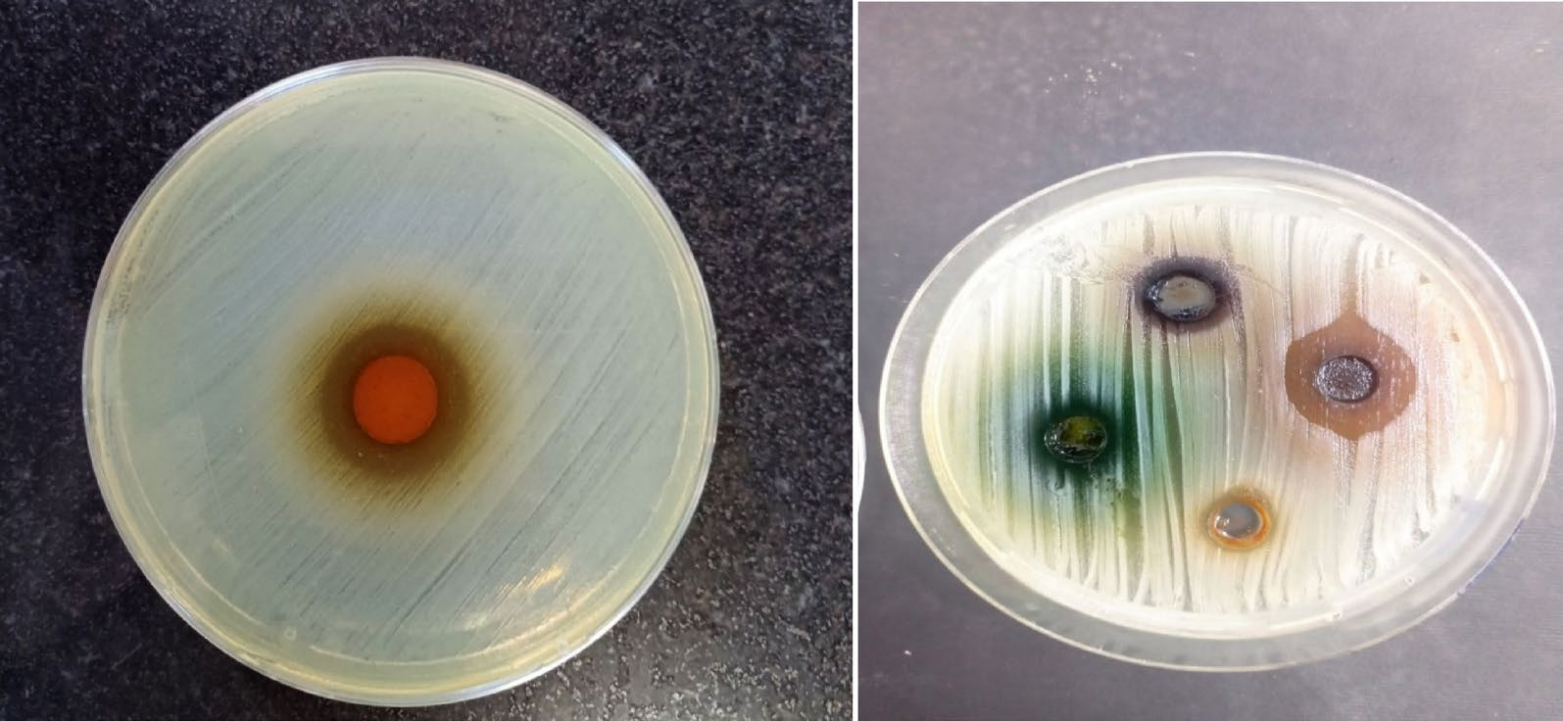
Effect of *the Ziziphus* fraction on *Pseudomonas aeruginosa* clinical isolates

The ZJ fraction showed antimicrobial activity, producing inhibition zones ranging from 18 to 22 mm on Mueller–Hinton agar (MHA). Bacteria were grown with an adjusted inoculum of 0.5 McFarland of the isolated *Pseudomonas aeruginosa*, and then incubated at 37 °C for 24 h. The diameter of the inhibition zone was measured, emphasizing the effect of the fraction, as shown in Fig. 2 with average zone size of 18 to 22 mm.

### Determination of minimum inhibitory concentration of ZJ fraction

The minimum inhibitory concentration (MIC) of the aqueous ZJ fraction was evaluated for the three Pseudomonas isolates (C1, C2, and C3). All three isolates demonstrated consistent MIC values of 1.56 mg/mL, suggesting a broad inhibitory effect of the fraction on resistant strains.

### Sub-inhibitory concentration of ZJ fraction on production of biofilm

At sub-MIC levels (0.78 mg/mL), the ZJ fraction significantly inhibited biofilm formation by MDR *P. aeruginosa*. Isolates C1, C2, and C3 exhibited 70%, 79%, and 61% reduction, respectively, compared to the untreated controls (p ≤ 0.01, n = 3). The results are shown in Fig. 6.

### Influence of ZJ fraction on lasI, rh1I, and rh1R genes expression levels

#### lasI gene

**Fig. 3:**
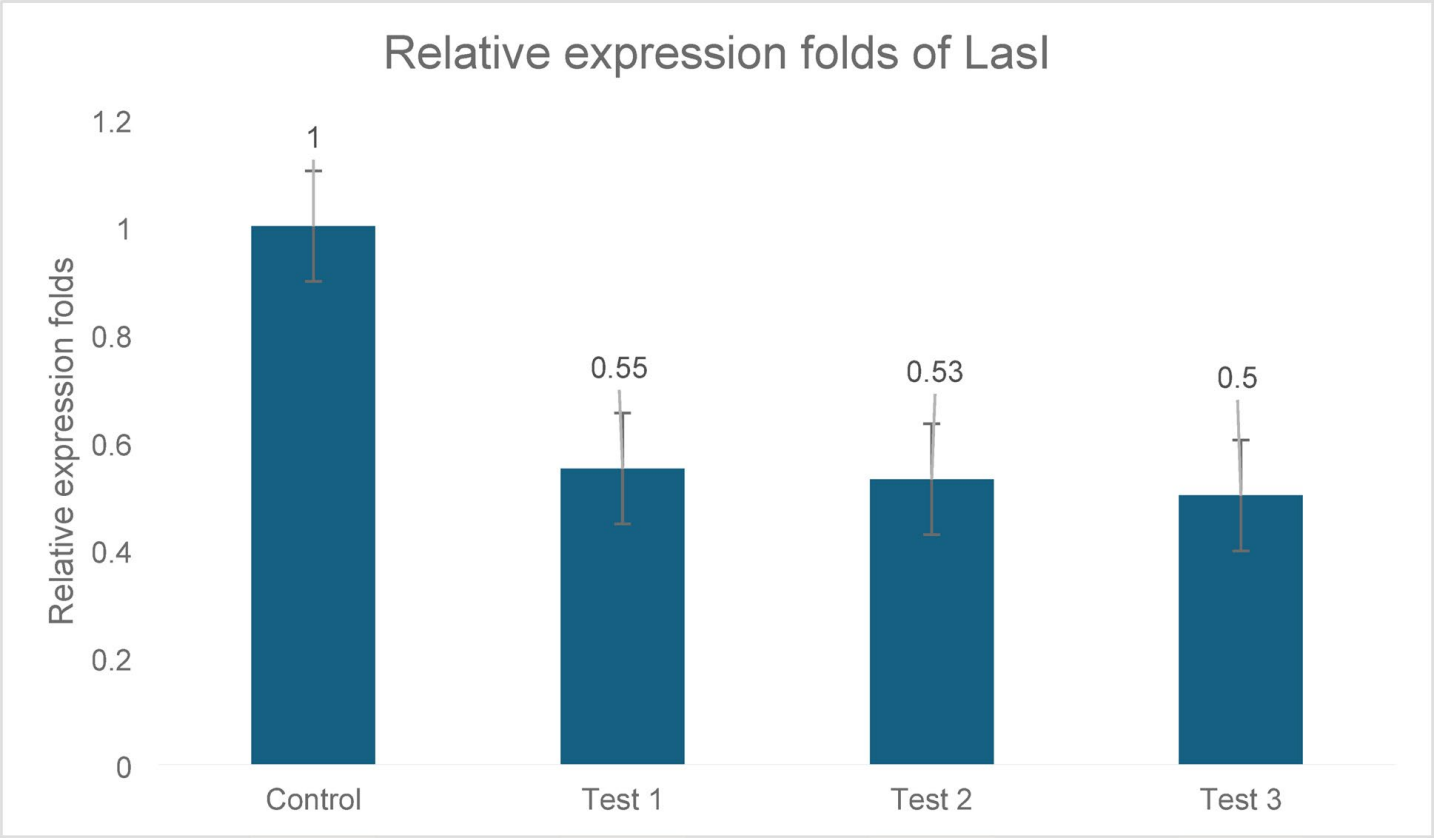
Expression analysis of *lasI* in three *P. aeruginosa* clinical isolates (C1, C2, and C3) following treatment with ZJ fraction. *LasI* significantly inhibited gene expression by 45% and 50% in trials 1, 2, and 3, respectively. This reduction was statistically significant compared to that in the untreated control (p ≤ 0.05). Gene expression levels were normalized to those of 5S rRNA

Treatment with the ZJ fraction led to significant downregulation of *lasI*: 45%, 47%, and 50% inhibition in C1, C2, and C3, respectively. All gene expression levels were normalized to 5S rRNA and showed statistically significant differences compared with the control (p ≤ 0.05), as shown in Fig. 3. All tests were normalized to the 5S rRNA gene.

#### rh1I gene

**Fig. 4:**
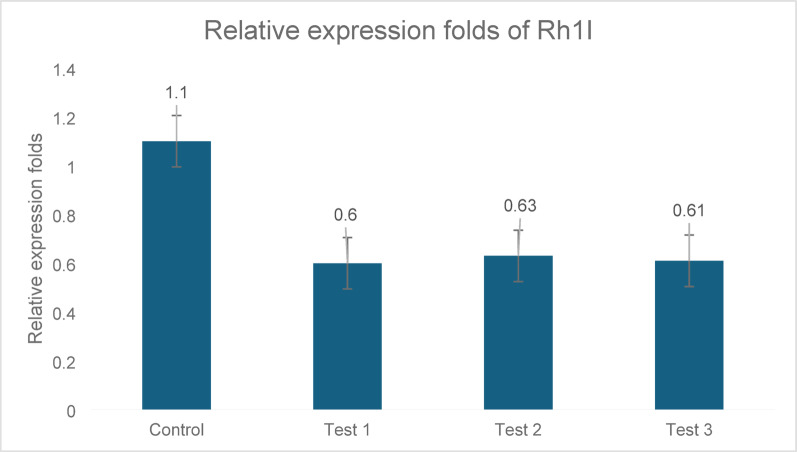
Expression analysis of *rh1I* in three *P. aeruginosa* clinical isolates (C1, C2, and C3) following treatment with ZJ fraction. *rh1I* significantly inhibited gene expression by 45.4%, 42.7%, and 44.5% in trials 1, 2, and 3, respectively. This reduction was statistically significant compared to that in the untreated control (p ≤ 0.05). Gene expression levels were normalized to those of 5S rRNA

*rh1I* expression was inhibited by 45.4%, 42.7%, and 44.5% in the three tested strains. Significance was confirmed using statistically significant differences compared with the control (p ≤ 0.05), (p ≤ 0.05), and data normalized to the 5S rRNA gene (Fig. 4).

#### rh1R gene

**Fig. 5:**
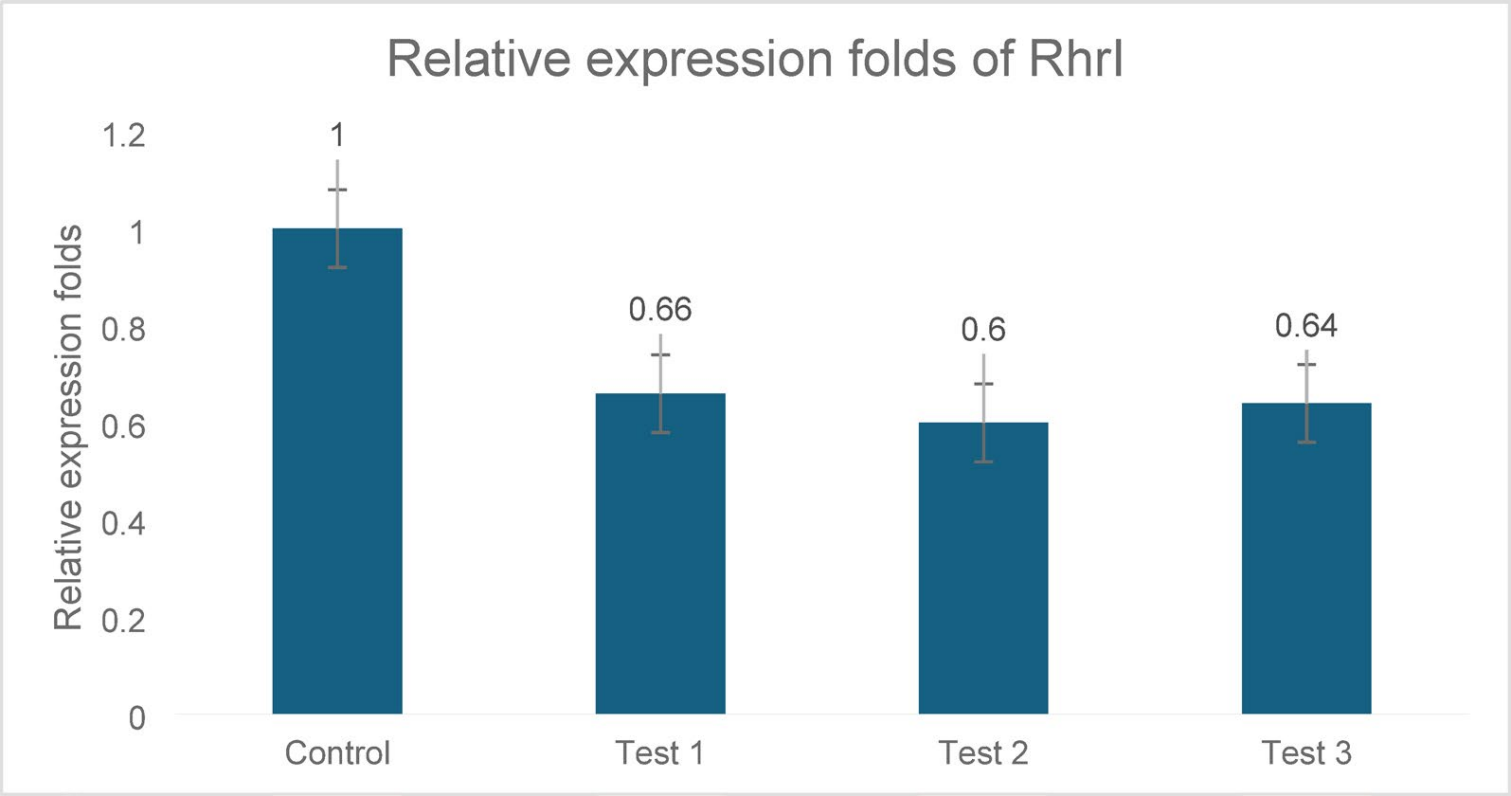
Expression analysis of *rhlR* in three *P. aeruginosa* clinical isolates (C1, C2, and C3) following treatment with ZJ fraction. *rhlR* significantly inhibited gene expression by 34%, 40%, and 36% in trials 1, 2, and 3, respectively. This reduction was statistically significant compared to that in the untreated control (p ≤ 0.05). Gene expression levels were normalized to those of 5S rRNA

*rhlR* showed reduced expression by 34%, 40%, and 36% in C1, C2, and C3, respectively. A statistically significant difference was observed (p ≤ 0.05), as shown in Fig. 5.

Significant reductions in virulence gene expression and biofilm formation were observed in the treated groups compared to the untreated controls (ANOVA, p < 0.001; Tukey's post-hoc test, p < 0.01 for all treated groups). The effect size was large (η^2^ = 0.35), indicating a strong effect of the ZJ extract on biofilm inhibition.

## Discussion

Over the past few decades, healthcare-associated infections, particularly those involving wounds and burns, have continued to pose a significant global challenge. Concurrently, the widespread and often unregulated use of antibiotics, especially in developing regions, has contributed to a notable rise in antibiotic resistance (Mohamed et al. 2022). Escalating resistance to current antimicrobials and antibiotics has necessitated the urgent development of novel, safe, and efficacious products with potent antimicrobial capabilities (Al-Majmaie et al. 2021).

*P. aeruginosa* stands as a primary contributor to healthcare-associated infections (HAI). The ramifications of untreated infections can be dire, potentially progressing to sepsis and ultimately to septic shock, which is considered fatal without appropriate medical treatment. The formidable resistance of this bacterium to antibiotics, coupled with the toxicity of colistin, a last-line antibiotic for *Pseudomonas* infections, has prompted the exploration of alternative therapeutic approaches.

The establishment of a biofilm at the site of a wound and burns is critical. Because of the difficulties of treating and getting rid of biofilms, all efforts were implemented to develop a new approach and a new drug that can interact with biofilms to accelerate the remedial procedure (Hurley et al. 2012; Agrawal et al. 2023).

Since ancient times, there has been a widespread belief in the medicinal efficacy of plants, with claims that they can cure diseases, treat wounds, and alleviate burns. Empirical evidence has substantiated this belief across various plant species, seeds, and herbal extracts. Studies indicate that plant extracts may serve as promising antimicrobial agents for treating *P. aeruginosa* healthcare-associated infections (HAI) (Alam et al. 2020).

Plants are abundant sources of primary and secondary metabolites. For cellular function processes, primary metabolites are generally characterized by high-molecular-weight compounds, such as carbohydrates, proteins, and nucleic acids (Bocso et al. 2022; Nazeam et al. 2022). In contrast, secondary metabolites are predominantly low-molecular-weight compounds that can vary widely in structure (Zandavar and Babazad 2023). Secondary metabolites in plants serve primarily as defensive mechanisms, providing protection against various threats such as predators, plant pathogens, insects, and animals (Mitra et al. 2023). Plants detect bacteria using pathogen-associated molecular patterns (PAMPs) or pathogen effectors (Gorlenko et al. 2020; Keita et al. 2022).

Given their crucial role in plant defense, secondary metabolites have garnered significant attention owing to their potential antimicrobial properties. Their diverse chemical structures and distinct modes of action make them promising candidates for combating microbial infections (Šovljanski et al. 2023). The purification of ZJ secondary metabolites was based on molecular weight variations between the plant metabolites. Following the addition of absolute ethanol to the aqueous extract, the high molecular weight fraction was precipitated and discarded (Nazeam et al. 2024). The filtrate containing low molecular weight secondary metabolites was concentrated and analyzed using LC–MS/MS for metabolite identification.

Plant phenolic compounds have shown promising antimicrobial effects against drug-resistant bacteria (Minich et al. 2022). LC–MS/MS-MRM analysis revealed that the most abundant compounds were 3,4-dihydroxybenzoic, gallic, syringic**,** chlorogenic, and ferulic acids. Our findings are consistent with those of previous studies that have highlighted the antimicrobial properties of plant-derived phenolic compounds.

In this study, 100 clinical *P. aeruginosa* isolates demonstrated biofilm production capabilities compared to the control, with high levels of antibiotic resistance (100% resistance to ticarcillin acid and piperacillin). Furthermore, these strains exhibited extended resistance to ceftazidime, amikacin, and cefepime. Consequently, these isolates demonstrated resistance to a wide range of commonly used antimicrobials. The study determined sub-minimum inhibitory concentration of *Z. jujuba* (ZJ) aqueous fraction and evaluated its effect on virulence genes in *P. aeruginosa* biofilm formation.

Analyses were conducted at half-MIC for both treated and control cells. The findings were promising, demonstrating a statistically significant difference between the untreated control group and *P. aeruginosa* isolates subjected to ZJ treatment.

Biological metal-chelating agents are recognized for their antimicrobial capabilities, primarily through the sequestration of vital metal ions such as iron, zinc, and copper. These metal ions are essential for microbial enzymatic function, DNA replication, and protection against oxidative stress (Banin et al. 2006). The absence of these metals disrupts microbial metabolic pathways, induces oxidative damage, and weakens cell membrane integrity, ultimately hindering microbial proliferation and causing cell death (Touati 2000). Notably, 3,4-dihydroxybenzoic acid, the most abundant compound identified in the ZJ fraction, has been characterized as a potent biological metal-chelator (Friggeri et al. 2015). This chelating property was likely responsible for the antimicrobial activity observed in this fraction. A previous study demonstrated that bandages made from nanofibers containing 2,3-dihydroxybenzoic acid (DHBA) could serve as an alternative therapy for *P. aeruginosa* skin infections. A study found that when *P. aeruginosa* Xen 5 was exposed to DHBA for 8 h, biofilm formation was reduced by approximately 75%, and the bacteria exhibited increased motility (Ahire et al. 2014).

Gallic acid (GA), a prominent plant polyphenol, has been recognized for its wide range of health benefits, particularly in combating bacterial and viral infections. Studies have demonstrated that GA impedes bacterial proliferation through several mechanisms, including disruption of membrane integrity, interference with bacterial metabolic processes, and prevention of biofilm formation (Keyvani-Ghamsari et al. 2023). Research has shown that Gallic acid impedes the initial attachment of *P. aeruginosa* and influences its ability to form biofilms (Wang et al. 2021). The minimum bactericidal concentration of *P. aeruginosa* to inhibit bacterial motility is 500 μg ml^−1^ for gallic acid (GA) and ferulic acid (FA) to inhibit bacterial motility (Borges et al. 2012). Additionally, GA has been found to convert ampicillin-resistant cells, both in planktonic and biofilm states, into highly susceptible cells by causing membrane damage and facilitating increased drug accumulation (Kosuru et al. 2018).

Syringic acid inhibited *S. epidermidis* biofilm formation in a concentration-dependent manner, and at its highest concentration, biofilm mass was reduced by 70% (Minich et al. 2022). Chlorogenic acid (CA), a caffeic acid ester, is a prominent phenolic compound found in various herbs. Research has shown that CA is a promising preservative for controlling foodborne illnesses associated with *P. aeruginosa*. This potential stems from the ability of CA to inflict damage to both the intracellular and outer membranes of cells, as well as its ability to interfere with cellular metabolism. These effects lead to the death of the bacterial cells (Su et al. 2019). Moreover, studies have shown that CA exhibits an antibiofilm effect on *P. aeruginosa,* and analysis using quantitative real-time PCR has indicated that CA disrupts the synthesis of signaling molecules and transcription regulators in the Las, Pqs, and Rhl systems (Sheikhy et al. 2014; Wang et al. 2023). Another study revealed its downregulation of *lasI*, *lasR*, *rhlI*, *rhlR*, *pqsA*, and *pqsR* QS‐ genes expression in *P*. *aeruginosa* (Xu et al. 2022).

In vivo studies on Caenorhabditis elegans and mouse infection models were conducted to explore the anti-virulence ability of CA. This finding indicates that CA prolonged the survival duration and diminished the amount of *P. aeruginosa* within the nematode intestine. Additionally, in a murine wound model, groups treated with CA exhibited an enhanced rate of healing, and the bacterial population at the wound site was reduced following CA administration (Wang et al. 2019).

Ferulic acid (FA) was found to suppress the generation of quorum sensing-controlled virulence elements in *P. aeruginosa*, including pyocyanin production, biofilm formation, and swarming behavior (Ugurlu et al. 2016). Moreover, FA exhibited inhibitory effect, higher than 40%, on most of the lasR, rhlR, aprA, and lasB genes in two *P. aeruginosa* strains, BAA-47 and 27,853 (Velasco et al. 2024).

**Fig. 6:**
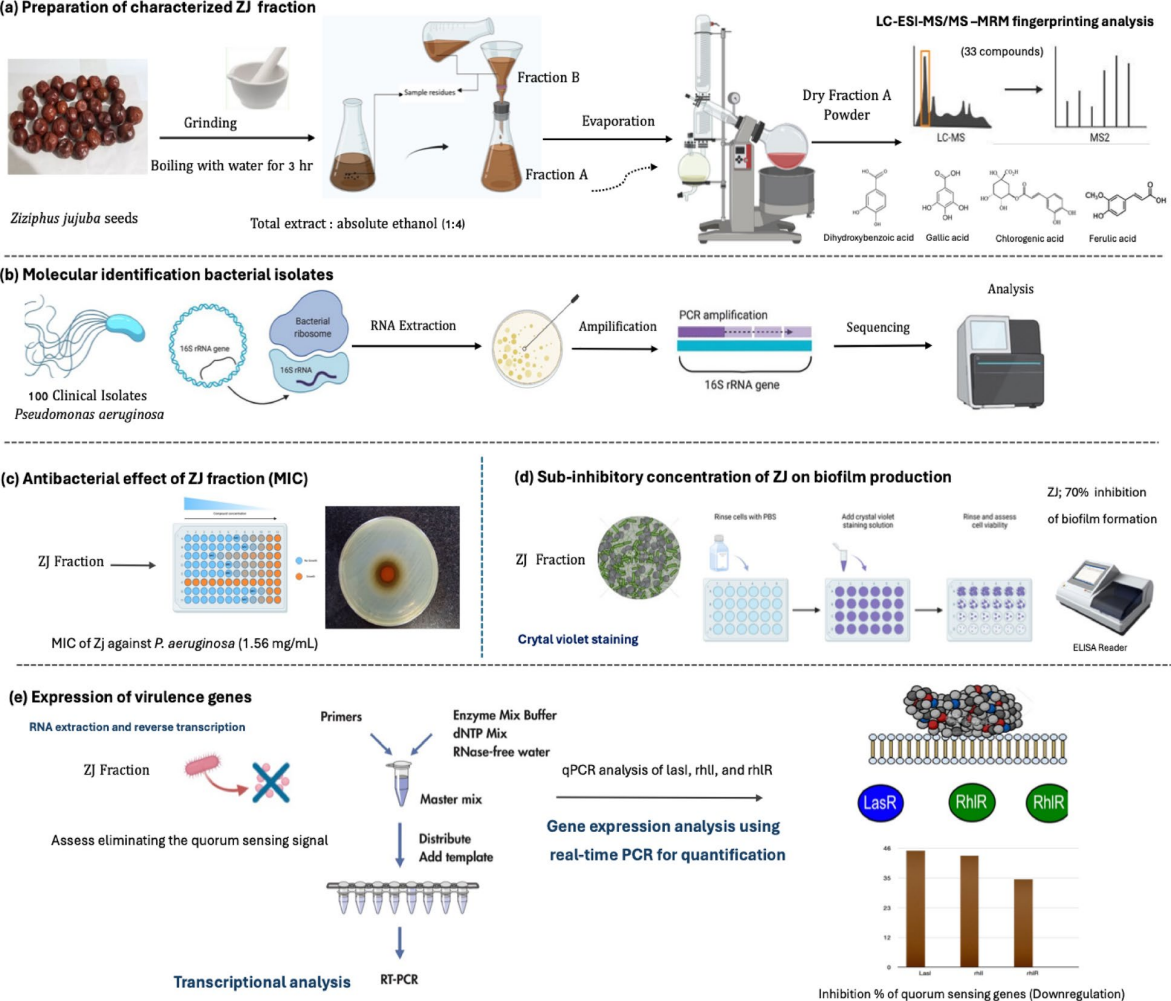
Visual methodology diagram of the experimental workflow

In summary, the ZJ fraction exhibited promising anti-*Pseudomonas aeruginosa* activity by targeting key quorum sensing (QS) genetic determinants, as demonstrated through both phenotypic and genotypic analyses. This effect is primarily attributed to the downregulation of *lasI*, *rhlI*, and *rhlR*, which disrupts the QS-regulated pathways critical for biofilm development (Fig. 6). Inhibiting these QS genes not only weakens biofilm formation and reduces bacterial virulence but also enhances antibiotic susceptibility, attenuates immune evasion strategies, and diminishes the pathogen’s ability to spread (Tuon et al. 2022). Furthermore, LC–ESI–MS/MS profiling revealed a rich spectrum of phenolic compounds, reinforcing the therapeutic potential of this natural product, and supporting its feasibility as a cost-effective alternative to conventional antibiotics.

While the antimicrobial properties of ZJ are compelling, it is imperative to acknowledge certain limitations. The study was conducted in vitro, which, although it offers valuable insights, does not fully replicate the complex host–pathogen interactions that occur in vivo. Furthermore, this study did not investigate the pharmacokinetics, toxicity, or potential immunomodulatory effects of the ZJ fraction, which are essential for assessing its therapeutic potential.

To address these gaps, future research should incorporate in vivo models to validate bioavailability, pharmacodynamic properties, and biosafety profiles. Additionally, comparative studies with other clinically relevant biofilm-forming pathogens, such as *Staphylococcus aureus* and *Klebsiella pneumoniae*, could enhance the applicability of the ZJ fraction. Mechanistic studies exploring its influence on QS-regulated virulence factors such as elastase, pyocyanin, and rhamnolipid production will also deepen the molecular understanding. Integrating omics-based approaches, particularly proteomics, metabolomics, and comprehensive transcriptomics, will offer a systems-level perspective on its antimicrobial action.

In terms of practical implications, the broader significance of this study lies in positioning plant-derived QS inhibitors, such as the ZJ fraction, as viable tools for combating antimicrobial resistance (AMR), particularly in infections where biofilms play a central role. The potential synergistic effects with existing antibiotics also open new avenues for combination therapies aimed at re-sensitizing resistant bacterial strains.

To provide a clear roadmap for clinical translation, we recommend a phased approach.

- Preclinical Phase: Perform in vivo studies to assess the efficacy and toxicity using appropriate animal models.
- Mechanistic Elucidation: c-di-GMP quantification assays and QS reporter systems were used to verify the disruption of the signaling pathways.
- Formulation Development: Create appropriate delivery systems using a standardized fraction, such as topical, inhalable, or oral formulations.
- Combination Studies: Investigate the synergistic interactions of antibiotics across diverse strains in both in vitro and in vivo settings.
- Pilot Clinical Trials: Design of early phase human trials to assess safety, tolerability, and preliminary efficacy.

Addressing these stages can expedite the transition of ZJ-based products from bench to bedside, potentially reducing infection burden, enhancing patient outcomes, and making a significant contribution to the global fight against resistant microbial pathogens.

## Data Availability

All data is available upon request.
